# Characteristics and clinical outcomes of patients presenting with advanced HIV disease in the “treat all” era: a retrospective cohort study from rural Rwanda

**DOI:** 10.1186/s12879-022-07692-w

**Published:** 2022-08-25

**Authors:** Gentille Musengimana, Jean Paul Umugisha, Placide Habinshuti, Todd Anderson, Geraldine Mukesharurema, Eric Remera, Jean D’Amour Ndahimana, Dale A. Barnhart

**Affiliations:** 1 Division of HIV/AIDs, Rwanda Biomedical Center, STIs and Viral Hepatitis, City of Kigali, Rwanda; 2 Partners in Heath, Inshuti Mu Buzima, Infectious Disease Program, City of Kigali, Rwanda; 3 Department of Global Health and Social Medicine Boston, Harvard Medical School, Boston, MA USA

**Keywords:** Rwanda, HIV, Delayed diagnosis, Treat all, Test and treat

## Abstract

**Background:**

In 2016 Rwanda adopted “treat all” where all patients with HIV are immediately eligible for ART regardless of disease progression. Despite widespread availability of treatment, it is unknown whether presentation with advanced HIV persists.

**Methods:**

We conducted a retrospective cohort among patients aged ≥ 15 who enrolled in care between July 2016 and July 2018 in three rural Rwandan districts. We estimated the prevalence of advanced HIV, defined as presenting with CD4 count < 200 cells/mm^3^ or WHO stage 3 or 4, and compared baseline characteristics of patients with and without advanced HIV. We compared cumulative incidences and time to events using Chi squared tests and Cox proportional hazards models, respectively, for (a) viral load tests; (b) viral suppression; (c) death; and (d) treatment failure (a composite of death, lost to follow up, or virologic failure).

**Results:**

Among 957 patients, 105 (11.0%) presented with advanced HIV. These patients were significantly more likely to have low body mass index, come from Burera district, be older, and be identified through inpatient settings rather than through voluntary or prenatal testing. Patients with advanced HIV had significantly higher risks of death at 12-months (9.5% vs 1.5%, p < 0.001) and 18-months (10.5% vs 1.9%, p < 0.001) and significantly higher risk of treatment failure at 12-months (21.9% vs. 14.2%, p = 0.037). After adjusting for confounders, patients with advanced HIV had still higher rates of death (adjusted Hazard ratio [aHR] = 4.4, 95% CI: 1.9, 10.2, p < 0.001) and treatment failure (aHR = 1.7, 95% CI: 1.1, 2.5, p = 0.017), but no difference in viral load testing (aHR = 1.1, 95% CI: 0.8, 1.5, p = 0.442) or viral suppression (aHR = 1.0, 95% CI: 0.8, 1.4, p = 0.949). When allowing for the hazard ratio to vary over time, patients with advanced HIV experienced elevated rates of treatment failure in the first six of enrollment, but not after nine months.

**Conclusion:**

Presenting with advanced HIV remains common and is still associated with poor patient outcomes. Sensitization of the community to the benefits of early ART initiation, identification of patients with advanced HIV, and holistic support programs for the first 6 months of treatment may be needed to improve outcomes.

## Background

In 2016, the World Health Organization’s (WHO) guidelines changed from antiretroviral treatment (ART) initiation based on CD4 count thresholds to a “treat all” strategy in which patients are eligible to initiate ART regardless of their CD4 count [1]. Studies have shown clear benefits of starting ART while patients are still asymptomatic [1–4]. The “treat all strategy” was designed to be accompanied with widespread access to HIV testing and was hypothesized to allow patients to benefit from early ART initiation through a variety of mechanisms, including providing ethical justification and personal motivation for routine asymptomatic testing, reducing stigma surrounding HIV, simplifying pre-initiation clinical procedures, and minimizing pre-ART loss to follow-up [5–8]. However, evidence from different African countries suggests a significant prevalence of advanced HIV among ART patients presenting for care even in the treat all era [9–11]. Despite efforts to scale up ART coverage over the past decade, in 2020 only 73% of the people living with HIV had access to ART and 680,000 people died from AIDS-related illness [12].

In Rwanda, the national HIV program has successfully decentralized access to free HIV services and by 2013 had created a national network of over 450 health facilities, almost all of which (97%) offered HIV testing and the vast majority of which (89%) offered ART with no cost required from patients [13]. In July 2016, Rwanda adopted the “treat all” strategy [14]. Subsequently, Rwanda has reached two of the three 2020 UNAIDs 90–90–90 targets, with 83.8% of people who live with HIV knowing their status, 97.5% of those who know their status being on ART treatment and 90.1% of those on treatment being virally suppressed [15]. However, in Rwanda, little is known about HIV positive patients presenting with advanced HIV since the start of “treat all”. This study aims to estimate the prevalence of patients presenting for treatment with advanced HIV after the start of the treat all era (July 2016 to July 2018) within health facilities in three rural Rwandan districts and compare clinical outcomes among patents presenting with and without advanced HIV.

## Methods

### Setting

The study was conducted in the catchment areas of Rwinkwavu, Kirehe and Butaro district hospitals, which include 43 affiliated health centers. Rwinkwavu and Kirehe are in rural Eastern province while Butaro is in the rural Northern Province of Rwanda. The HIV program in these areas is implemented by the Rwandan Ministry of Health (MoH) through Rwanda Biomedical Center (RBC) and receives technical and financial support from Partners In Health/Inshuti Mu Buzima (PIH/IMB), which is an international nongovernmental organization.

In accordance with the current Rwandan national guidelines, patients with HIV are eligible to receive free ART regardless of their baseline CD4 count or WHO stage and are enrolled in the Differentiated Service Delivery Model (DSDM) [14]. Through the DSDM model, new HIV patients receive monthly ART prescriptions and quarterly clinical visits until they have two suppressed consecutive viral load (VL) measurements < 200 copies/ml, at which point they transition to quarterly ART prescriptions and biannual clinical visits. Because national guidelines recommend same-date treatment initiation for most patients and stipulate that VL testing should occur 6 and 18 months after treatment initiation, the earliest this transition to less frequent service delivery can occur is 18 months after enrollment in care. In addition to the national standard of care, elderly or disabled patients in PIH/IMB-supported sites receive community-based accompaniment, which has been shown to improve patient outcomes [16]. PIH/IMB-supported sites also receive system strengthening. Nurses and doctor mentors at PIH/IMB-supported sites are provided with onsite mentorship on guidelines and management of complicated cases, and patients receive financial support such as transport stipends and food packages for prevention of mother to child transmission (PMTCT) patients.

### Study design and population

We conducted a retrospective cohort study to compare patients with HIV who presented for treatment with advanced HIV to those who presented without advanced HIV in PIH/IMB-supported health facilities. We included all patients with HIV aged 15 years and above who entered the HIV program between July 1st 2016 and July 31st 2018. Patients with neither a baseline CD4 count nor a baseline WHO stage reported could not be classified as presenting with or without advanced HIV and were excluded from the study.

### Sources of data

We used routinely collected data extracted from electronic medical record (EMR). Variables extracted or derived from the EMR included district; age; sex; distance to health facility; Body Mass Index (BMI) at baseline categorized as < 16, 16–18.5, and > 18.5; WHO stage at baseline, CD4 count at baseline, and method of enrolment into the HIV program, categorized as voluntary counselling and testing (VCT), PMTCT, inpatient and tuberculosis patients, and outpatient patients. For BMI, WHO stage, and CD4 count, the earliest measurement within 180 days of enrollment was considered the baseline measurement. Additional data extracted from the EMR included date of enrollment into the program, date of ART initiation, dates of scheduled and observed patient visits, viral load results, dates of collection for viral load results, and date of death or exit from the HIV program.

### Exposure and outcome variables

Because our analysis used existing medical records, many patients were missing information on either CD4 count or WHO stage. Consequently, patients presenting with CD4 count < 200 cells/mm^3^ at enrollment or WHO stage 3 and 4 at enrollment were classified as presenting with advanced HIV [17]. The remaining patients were classified as presenting without advanced HIV if they had documentation of either (a) both CD4 count > 200 cells/mm^3^ and baseline WHO stage 1 or 2, or (b) baseline CD4 count > 200 cells/mm^3^ at enrollment and no documentation of WHO stage 3 or 4, or (c) baseline WHO stage 1 or 2 and no documentation of CD4 count.

In Rwanda, treatment guidelines recommend to provide same-day treatment initiation for HIV when possible and within the two weeks of diagnosis for almost all patients and viral load testing 6- and 18-months after treatment initiation. As a conservative measure of adherence to these guidelines, we assessed the proportion of patients who initiated ART within 14 days of presentation and the proportion of patient who received an initial viral load test within 9 months (270 days) of presentation. Among patients who received an initial viral load test within 270 days of enrollment, we also reported the proportion who were virally suppressed, defined as < 200 copies/ml. We also reported the 12- and 18-month cumulative incidence of death; loss to follow-up and virological failure, and treatment failure. Lost to follow-up was assumed to occur on the last observed visit date if the patient had no recorded clinical visits for > 3 months following their last scheduled appointment or > 7 months after the last visit date. Virological failure was defined as observing viral load ≥ 200 copies/ml among those who had previously achieved viral suppression. We defined treatment failure as a composite outcome that included death, loss to follow-up, or virological failure. In rural Rwanda, where patients rely almost exclusively on the local public hospitals and health centers for access to HIV treatment and deaths are sometimes unreported in the EMR, these outcomes are closely correlated. For example, patients who have become loss to follow up are unlikely to be accessing any HIV treatment at all, are at risk of virologic failure, and may be dead. Our composite outcome of treatment failure reflects the fact that any of these events are of clinical concern.

We also calculated four time-to-event outcomes: time to viral load test, time to viral suppression, time to death, and time to treatment failure. Time to first viral load test and time to viral suppression, which was defined as the time to the first viral load measurement < 200 copies/ml, were both censored by death, loss to follow-up, or transferring out of the program. Time to death was censored by loss to follow-up or transferring out of the program. Time to treatment failure was defined the earliest occurrence of death, loss to follow-up, or virological failure and was censored by transfer out of the program. If a patient experienced an event of interest or a censoring event on their day of enrollment, we set the time to event to 0.5 days to ensure that these individuals were not excluded from analysis. All time to event outcomes were administratively censored at 21 months (630 days). This time frame was chosen to allow patients a reasonable opportunity to complete their second viral load test, which should occur approximately 18 months after treatment initiation according to national guidelines but is often delayed in practice.

### Statistical analysis

We described and compared demographic characteristics, clinical characteristics, and clinical outcomes of patients presenting with advanced and non-advanced HIV using frequencies and Chi-squared tests or Fisher’s exact tests for variables with cell counts < 5. Due to small cell counts, the VCT and PMTCT categories were collapsed in regression analyses. We created missing indicator categories to account for missing data. We compared time to event for our four main outcomes among patients presenting with and without advanced HIV using Kaplan–Meier curves and log-rank tests. For each outcome, we fit three Cox proportional hazards models to estimate hazard ratios and corresponding 95% confidence intervals (CIs) for the association between presenting with advanced HIV and experiencing the outcome of interest. The first model was unadjusted. The second model adjusted for baseline demographic characteristics, including district, age, sex, and distance from the health facility, that could be common causes of both presenting with advanced HIV and experiencing treatment failures and were therefore considered to be potential confounders [18]. Two variables, method enrollment and BMI category, could have been considered to be either confounders or as mediating variables. For example, low BMI could either be a marker for low socioeconomic status, and therefore a likely confounder that should be adjusted for, or consequence of advanced HIV, and therefore a mediating variable that should not be adjusted for. To ensure our findings were not sensitive to assumptions regarding whether these variables were confounders or mediators, we present a third set of maximally adjusted models that included all the baseline demographics from our adjusted analysis as well as method enrollment and BMI category. Baseline CD4 count and WHO stage were not considered to be possible confounders in our model because they were used to define our primary exposure of interest, presentation with advanced HIV.

For each outcome and each model, we assessed whether the proportional hazards assumption held using a global test of Schoenfeld residuals. If we observed a significant association on the global test, we assessed the association between each covariate’s residuals and time to identify which covariates violated the proportional hazards assumptions. The global test of Schoenfeld residuals was only observed to be statistically significant for one model, which was the unadjusted association between presentation with advanced HIV and time to treatment failure. Despite the violation of proportional hazards, we present this unadjusted hazard ratio as it is still interpretable as an approximation for the average incidence rate ratio comparing treatment failure among patients presenting for care with and without advanced HIV over 630 days of follow-up [19]. However, to understand how the hazard ratio for treatment failure changed over time, we also fit an additional Cox Proportional Hazards model for the association between advanced HIV and treatment failure model that adjusted for baseline demographics and included a time-varying interaction term between advanced HIV status and linear time centered at 180 days. This interaction term was selected to ensure appropriately distributed Schoenfeld residuals. We used this model to calculate hazard ratios and 95% CIs at 90-day intervals. All analyses were conducted in Stata version 15.1 [20].

## Results

Of the 1129 patients aged 15 years or older who enrolled in care for HIV at a PIH-IMB supported site between July 2016 and July 2018, 957 participants had data on baseline CD4 or WHO stage. Among these 957 patients, 105 (11.0%) presented with advanced HIV and 852 (89.0%) presented with non-advanced HIV (Table 1). Advanced HIV patients were more likely than non-advanced to be found in Burera district (46.7% vs. 22.4%). Older age was significantly associated with advanced HIV disease, with 39.0% of advanced HIV patients being ≥ 45 compared to 19.0% of non-advanced. There were no statistically significant differences in sex or distance to health facility between the two groups. Compared to non-advanced HIV patients, advanced HIV patients were more likely to be underweight (BMI < 18.5, 20.9%. vs 9.6%) and to be severely underweight (BMI < 16, 8.6% vs 1.5%). As expected, based on our definition of advanced HIV, most advanced HIV were classified as WHO stage 3 or 4 (56.2%) and 58.1% had baseline CD4 count < 200 cells/mm^3^. Although the majority (51.6%) of non-advanced HIV were identified through VCT or PMTCT, only 20.0% of advanced HIV were identified through these venues.

**Table 1:** Sociodemographic and clinical characteristics of patients presenting with and without advanced HIV (N = 957)

	Advanced HIV (N = 105)^a^		Non-advanced HIV (N = 852)		p-value
	N	%	N	%	
District					< 0.001
Kayonza	34	32.4	298	35.0	
Kirehe	22	21.0	363	42.6	
Burera	49	46.7	191	22.4	
Age at baseline					< 0.001
15–24	13	12.4	187	22.0	
25–34	19	18.1	324	38.0	
35–44	32	30.5	179	21.0	
≥ 45	41	39.1	162	19.0	
Sex					0.090
Female	61	58.1	566	66.4	
Male	44	41.9	286	33.6	
Distance to health facility (km)					0.300
0–2	19	18.1	221	25.9	
> 2–5	42	40.0	308	36.2	
> 5	23	21.9	188	22.1	
Missing	21	20.0	135	15.9	
BMI category^c^					< 0.001
< 16	9	8.6	13	1.5	
16–18.5	13	12.4	69	8.1	
> 18.5	40	38.1	443	52.0	
Missing	43	41.0	327	38.4	
WHO stage					< 0.001^b^
Stage1	25	23.8	713	83.7	
Stage2	9	8.6	86	10.1	
Stage3	48	45.7	0	0.0	
Stage4	11	10.5	0	0.0	
Missing	12	11.4	53	6.2	
CD4 count (cells/mm^3^)					< 0.001^b^
< 200	61	58.1	0	0.0	
200–350	6	5.7	64	7.5	
351–499	4	3.8	64	7.5	
500 and above	2	1.9	189	22.2	
Missing	32	30.5	535	62.8	
Method of enrollment					< 0.001^b^
VCT^c^	20	19.1	338	39.7	
PMTCT^c^	1	1.0	102	12.0	
Inpatient or tuberculosis	11	10.5	10	1.2	
Outpatient	15	14.3	70	8.2	
Other/missing	58	55.2	332	39.0	

^a^Advanced HIV  were defined as those who presented to care with CD4 < 200 or WHO Stage 3 and 4

^b^Fisher's exact test was used due to small cell count

^c^VCT: Voluntary Counselling and Testing; PMTCT: Prevention of Mother to Child Transmission, BMI: Body Mass Index

When comparing patients with and without advanced HIV, there were no statistically significant differences in initiating ART within 14 days, receipt of a viral load test within 9 months or viral suppression at first test (Table 2). However, in both groups less than 70% of patients-initiated care within two weeks of enrollment and less that 50% received an initial viral load test within 9 months of treatment. Patients presenting with advanced HIV were more likely to experience treatment failure in the first 12 months (21.9% vs 14.2%, p = 0.037) and to experience death in their first 12 months (9.5% vs 1.5%, p < 0.001). By 18 months, only the cumulative incidence of death remained different between the two groups (10.5% vs 1.9%, p < 0.001). There was no association between presenting with advanced HIV and loss to follow-up or virologic failure at 12 or 18 months (Table 2).

**Table 2:** Cumulative incidence of clinical outcomes among patients presenting with and without advanced HIV (N = 957)

	Advanced HIV (N = 105)		Non-advanced HIV (N = 852)		p-value
	N	%	N	%	
ART Initiation within 14 days	64	61.0	594	69.7	0.067
Receipt of a viral load test within 9 months	45	42.9	297	34.9	0.107
Viral load < 200 copies/ml at first test within 9 months (N = 342)	33	73.3	230	77.4	0.542
Treatment failure^a^ at 12 months	23	21.9	121	14.2	0.037
Dead at 12 months	10	9.5	13	1.5	< 0.001
Lost to follow-up at 12 months	14	13.3	106	12.4	0.795
Virological failure^b^ at 12 months	0	0.0	2	0.2	0.793^c^
Treatment failure^a^ at 18 months	26	24.8	163	19.1	0.171
Dead at 18 months	11	10.5	16	1.9	< 0.001
Lost to follow-up at 18 months	15	14.3	143	16.8	0.515
Virological failure^b^ at 18 months	1	1.0	4	0.5	0.441^c^

^a^Treatment failure was composite outcome that included death, loss to follow-up, or virological failure

^b^Virological failure which was defined as having a viral load ≥ 200 copies/ml among those who had previously achieved viral suppression

^c^Fisher's exact test was used due to small cell count

**Fig. 1:**
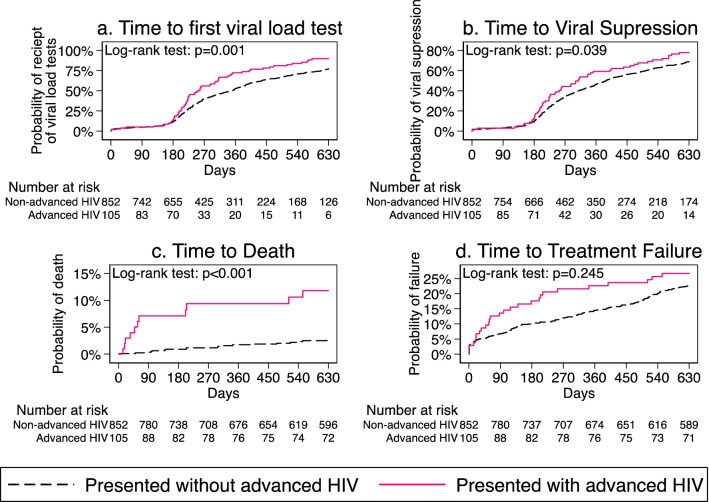
Kaplan–Meier curves comparing clinical outcomes among patients presenting with and without advanced HIV (N = 957)

**Fig. 2:**
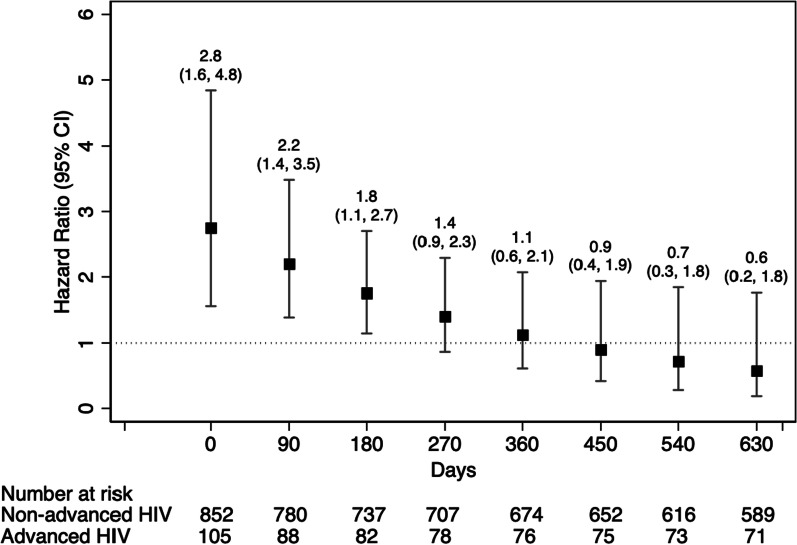
Time varying hazards ratio and 95% Cis for the association between presenting with advanced HIV and time to treatment failure. Hazard ratios were adjusted for district, age, sex, and distance to health facility (N = 957)

Patients presenting with advanced HIV had a significantly shorter time to first viral load test according to the log-rank test (Fig. 1a, p = 0.001) and the unadjusted Cox proportional hazards model (Table 3, HR = 1.5, 95% CI: 1.2, 2.0, p = 0.001), however, this association did not persist after adjustment for confounders. Similarly, advanced HIV was associated with shorter time to viral suppression in the log-rank test (Fig. 1b, p = 0.039) and the unadjusted Cox proportional hazards model (HR = 1.3, 95% CI: 1.0, 1.7, p = 0.040), but not after adjusting for confounders. However, patients presenting with advanced HIV had a much higher incidence of death in the log-rank test (Fig. 1c, p < 0.001) and the unadjusted Cox proportional hazards model (HR = 5.3, 95% CI: 2.5, 11.2, p < 0.001). This association persisted adjusting for demographic characteristics (aHR = 4.4, 95% CI: 1.9, 10.2, p < 0.001) and after adding BMI and method of enrollment to the model (aHR = 4.8, 95% CI: 2.0, 11.6, p = 0.001). Treatment failure was not associated with advanced HIV in the unadjusted in a log-rank test analyses (Fig. 1d, p < 0.245) but became significant after adjusting for demographic characteristics (aHR = 1.7, 95% CI: 1.1, 2.5, p = 0.017) and after adding BMI and method of enrollment to the model (aHR = 1.9, 95% CI: 1.2, 3.0, p = 0.005). When we allowed for the hazard ratio for the association between treatment failure and advanced HIV to vary over time, we observed that patients with advanced HIV experienced elevated rates of treatment failure in the first six of enrollment. However, by 9 months, the association between presenting with advanced HIV and treatment failure was no longer significant (Fig. 2).

**Table 3:** Hazard ratios from Cox Proportional Hazards models comparing the association between presenting with advanced HIV^a^ and time to and clinical outcomes^a^ (N = 957)

	Crude analysis			Adjusted analysis^b^			Maximally adjusted^c^		
	HR^f^	95% CI	p-value	aHR^f^	95% CI	p-value	aHR^f^	95% CI	p-value
First VL test	1.5	1.2, 2.0	0.001	1.2	0.9, 1.6	0.167	1.1	0.8, 1.5	0.442
First viral suppression	1.3	1.0, 1.7	0.040	1.1	0.8, 1.4	0.562	1.0	0.8, 1.4	0.949
Death	5.3	2.5, 11.2	< 0.001	4.4	1.9, 10.2	< 0.001	4.8	2.0, 11.6	0.001
Treatment failure^d^	1.3^e^	0.8, 1.9	0.247	1.7	1.1, 2.5	0.017	1.9	1.2, 3.0	0.005

^a^Defined as presenting to care with CD4 < 200 or WHO Stage 3 and 4

^b^Adjusted for district, age, sex, and distance to health facility

^c^Adjusted for district, age, sex, distance to health facility, BMI, and method of enrollment

^d^Treatment failure was composite outcome that included death, loss to follow-up, or virological failure where virological failure which was defined as having a viral load ≥ 200 copies/ml among those who had previously achieved viral suppression

^e^Proportional hazards assumption was violated for this model and the hazard ratio should be interpreted as the incidence rate ratio over the 630-day follow-up rather than an instantaneous hazard

^f^HR: Hazard ratio, aHR: adjusted Hazard ratio

## Discussion

Among patients presenting for care in PIH/IMB-supported health facilities between July 2016 and July 2018, 11.0% presented with advanced HIV. As has been reported elsewhere, older age and low BMI were associated with advanced HIV [21–24], although we did not observe the expected associations between presenting with advanced HIV and either male sex or distance to the health facility. In our study, Burera district had the highest proportion of patients with advanced HIV. The Butaro district hospital, located in Burera, is Rwanda’s main cancer referral hospital and provides treatment for many cancers associated with HIV, such as HPV-related cancers or Hodgkin’s lymphoma [25]. For example, previous research in Butaro revealed that over 30% of cervical cancer patients had HIV as comorbidity [26]. We hypothesize that the higher prevalence of patients with advanced HIV in Burera may indicate that many patients who develop HIV-related cancers as a result of living with advanced HIV are referred to Burera for cancer treatment and subsequently diagnosed with HIV.

The percentage of patients presenting with advanced HIV is lower than what has previously been reported by other researchers in Africa since the adoption of treat all, including in Senegal (71%), South Africa (22%-26%) and Botswana (24.7%) [9, 11, 27]. Although the prevalence of advanced HIV was lower in this study than reported in the literature, possibly due to Rwanda’s highly decentralized HIV services, patients presenting with advanced HIV still experienced death at approximately 4 times the rate for those without advanced HIV, translating a 12-month cumulative incidence of death of 9.5% and an 18-month cumulative incidence of death of 10.5%. Comparable risks of death among patients with advanced HIV were observed prior to the initiation of treat all in Uganda, Zimbabwe, Malawi, and Kenya [28]. Collectively, these studies and many others indicate patients with advanced HIV disease remain a vulnerable population in the treat all era [29, 30]. In our study, the prevalence of advanced HIV was relatively low, 11 out of 27 deaths (41%) occurred among those with advanced HIV. Providing targeted support to patients presenting with advanced HIV, particularly during the first 6-months of treatment when the risk of death, loss to follow-up or virologic failure is especially high, could substantially reduce HIV-related mortality.

Surprisingly, in our unadjusted analyses we observed time to viral load testing and viral suppression were faster among patients presenting with advanced HIV. This finding is likely the result of selection bias due to competing risks of death as death was associated with advanced HIV and because death was substantially more likely to occur among patients with advanced HIV in the first six months of treatment. Consequently, the sickest advanced HIV patients would have exited the cohort through death before becoming eligible for viral load testing. However, this association did not persist after adjusting for confounders, and ultimately our results suggest that patients with advanced HIV who survive and remain and treatment experience similar virologic outcomes as patients without advanced HIV. However, in both groups we observed suboptimal adherence to Rwanda’s national guidelines: Less than 70% of patients initiated care within two weeks of enrollment and less than 50% received their initial viral load test within 9 months of treatment. It was reported that newly-diagnosed PLWH in Rwanda, initiating ART rapidly under Treat All presents logistical and emotional challenges despite the perceived benefits [31] and viral load suppression was less among patients with a pre-ART CD4 count less than 200 cells/mm^3^ [32]. In general, these finding could be an indication of poor quality of care which need to be investigated further and addressed.

Our findings suggest multiple opportunities to improve HIV services. First, although Rwanda has decentralized HIV testing, there is a need to increase patient engagement with existing HIV testing services, which could include strategies such as expanded self-testing options, provision of HIV tests through the workplace or at the community, and increasing community awareness of that Undetectable = Untransmitable (“U = U)” among others [33]. Second, there is need to address barrier that prevent patients, both with and without advanced HIV, from initiating ART in the first 14 days. Previous research in Rwanda suggests that enhanced, trauma-informed counseling and stigma-reduction interventions could better support patients to initiate ART in a timely manner [31]. Third, we observed substantial missingness of baseline CD4 count information. However, CD4 remains an important biomarker for HIV patients’ management and care, and universal pre-ART CD4 count testing is needed to ensure an accurate diagnosis of those with advanced HIV disease [34]. Third, after patients presenting with advanced HIV are identified, they should receive the WHO-recommended package of care, including rapid ART initiation, screening for tuberculosis and cryptococcal meningitis, preemptive fluconazole treatment as well as cotrimoxazole and isoniazid prophylaxis [35, 36]. Fourth, psychosocial support, including food packages and Community-based Accompaniment (CBA), which had been previously been demonstrated to improve patient outcome at PIH/IMB-supported sites [37, 38] but were largely discontinued following the adoption of treat all, should be automatically expanded to those with advanced HIV. This targeted support should persist for the first 6 months of treatment, when the patients are at highest risk of treatment failure. Finally, there is need for further research to identify whether there are specific causes of death that could be prevented among late presenters.

Our study had some limitations: First, we used routinely collected data from EMR and some variable of interest, including cause of death and markers of socioeconomic status, were commonly missing from the EMR. Due COVID-related movement restrictions, we were unable to supplement the EMR with data from patients’ chart. In particular, missing CD4 and WHO stage information led us to use a definition of non-advanced HIV that was vulnerable to misclassification. We would have expected this bias would be expected to underestimate the true prevalence of advanced HIV disease and bias associations between advanced HIV disease and poor clinical outcomes towards the null. However, missing data could have also caused us to underreport timely ART initiation or timely viral load testing and over-report loss to follow-up. Second, our time-to-event analyses for death does not account for potential competing risks due to loss to follow-up, which is likely associated with death. If loss to follow-up were associated with advanced HIV, the analyses for these outcomes could have underestimated the associated between death and advanced HIV. However, we did not observe any associations between advanced HIV and loss to follow-up. Furthermore, the composite outcome of treatment failure included, viral suppression, death, and loss to follow-up, and is consequently not vulnerable to bias from competing events. Similarly, our time-to-event analyses for time to viral testing and time to viral suppression do not account for the competing risks of death, which, as previously discussed, could account for the surprising finding that time to viral load testing and viral suppression were faster among patients presenting with advanced HIV. Third, our analysis focuses on three rural districts that receive substantial support from an international NGO, so our results might not be generalizable elsewhere in the country. However, because of the additional support provided to these facilities, we would generally expect better HIV-related outcomes, including lower proportion of patients presenting for care with advanced HIV and lower rates of death and treatment failure, than in other rural Rwandan settings, suggesting this problem could be more widespread elsewhere.

## Conclusion

Despite adoption of the treat all, presentation for care with advanced HIV disease is still common in Rwanda and is still associated with poor treatment outcome. There is need for enhanced sensitization around the benefit of early HIV testing and availability of ART for all. In addition, patients presenting with advanced HIV may require additional support improve their treatment outcomes. More studies are needed to identify the specific causes of death among late presenters to help identity and prevent common causes of deaths.

## Data Availability

The datasets for this paper are not publicly available but are available from the corresponding author on reasonable request and approval from the IMB Research committee (imbrc@pih.org).
